# Two adjacent C-terminal mutations enable expression of aryl-alcohol oxidase from *Pleurotus eryngii* in *Pichia pastoris*

**DOI:** 10.1007/s00253-021-11585-4

**Published:** 2021-09-21

**Authors:** Nina Jankowski, Vlada B. Urlacher, Katja Koschorreck

**Affiliations:** Institute of Biochemistry, Heinrich-Heine-University Düsseldorf, Universitätsstraße 1, 40225 Düsseldorf, Germany

**Keywords:** *Pichia pastoris* (*Komagataella phaffii*), Aryl-alcohol oxidase, *Pleurotus eryngii*, Site-directed mutagenesis, Salt bridges, Gene copy number

## Abstract

Fungal aryl-alcohol oxidases (AAOs) are attractive biocatalysts because they selectively oxidize a broad range of aromatic and aliphatic allylic primary alcohols while yielding hydrogen peroxide as the only by-product. However, their use is hampered by challenging and often unsuccessful heterologous expression. Production of *Pe*AAO1 from *Pleurotus eryngii* ATCC 90787 in *Pichia pastoris* failed, while *Pe*AAO2 from *P. eryngii* P34 with an amino acid identity of 99% was expressed at high yields. By successively introducing mutations in *Pe*AAO1 to mimic the sequence of *Pe*AAO2, the double mutant *Pe*AAO1 ER with mutations K583E and Q584R was constructed, that was successfully expressed in *P. pastoris*. Functional expression was enhanced up to 155 U/l via further replacements D361N (variant NER) or V367A (variant AER). Fed-batch cultivation of recombinant *P. pastoris* yielded up to 116 mg/l of active variants. Glycosylated *Pe*AAO1 variants demonstrated high stability and catalytic efficiencies similar to *Pe*AAO2. Interestingly, *P. pastoris* expressing *Pe*AAO1 variant ER contained roughly 13 gene copies but showed similar volumetric activity as NER and AER with one to two gene copies and four times lower mRNA levels. Additional H-bonds and salt bridges introduced by mutations K583E and Q584R might facilitate heterologous expression by enhanced protein folding.

**Key points**

• *PeAAO1 not expressed in P. pastoris and PeAAO2 well-expressed in Pichia differ at 7 positions.*

• *Expression of PeAAO1 in P. pastoris achieved through mutagenesis based on PeAAO2 sequence.*

• *Combination of K583E and Q584R is essential for expression of PeAAO1 in P. pastoris.*

**Supplementary Information:**

The online version contains supplementary material available at 10.1007/s00253-021-11585-4.

## Introduction

Flavin-dependent oxidases build a diverse group of enzymes that have been successfully used in biocatalysis and biosensors (Dijkman et al. 2013). An important prerequisite for the application of these enzymes is their availability at high quantities. In this regard, heterologous expression in microbial hosts has been recognized as the most efficient approach which on the one hand, opens the way to high-scale processes and, on the other hand, when combined with protein engineering enables production and screening of mutants or mutant libraries to “tailor” the optimal biocatalyst for a specific purpose (Li and Cirino 2014). Aryl-alcohol oxidases (AAOs, EC 1.1.3.7) belong to flavin-dependent oxidases (Serrano et al. 2020). They contain a non-covalently bound FAD and catalyze the oxidation of primary aromatic and aliphatic allylic alcohols to the corresponding aldehydes, and if the *gem*-diol is formed, also to the corresponding acids (Ferreira et al. 2010; Guillén et al. 1992). AAOs are predominantly produced in wood-decaying fungi and secreted as glycoproteins. For their reactions, AAOs require only molecular oxygen and release hydrogen peroxide as the by-product (Guillén et al. 1992). Various studies have demonstrated the potential of AAOs for biotechnology (Urlacher and Koschorreck 2021). The most studied representative of this group is the AAO from *Pleurotus eryngii* ATCC 90787 (further designated as *Pe*AAO1), that among others was applied for the production of the flavor and fragrance compound *trans*-2-hexenal (de Almeida et al. 2019; van Schie et al. 2018). This enzyme was also used for the conversion of 5-hydroxymethylfurfural to 2,5-furandicarboxylic acid as precursor for bioplastics in multi-enzyme cascades (Carro et al. 2014; Serrano et al. 2019a). Furthermore, *Pe*AAO1 was engineered to oxidize secondary alcohols to facilitate kinetic deracemization (Serrano et al. 2019b; Viña-Gonzalez et al. 2019). *Pe*AAO1 has served as a model enzyme for numerous investigations providing insights into substrate spectrum, structural, and mechanistic properties of AAOs (Carro et al. 2017, 2018; Fernández et al. 2009; Ferreira et al. 2009, 2010; Guillén et al. 1992; Hernández-Ortega et al. 2012a, 2012b). However, heterologous expression of *Pe*AAO1 and AAOs in general is quite challenging and often unsuccessful or leads to only low amounts of active enzyme (Urlacher and Koschorreck 2021). For example, heterologous expression of *Pe*AAO1 in *Aspergillus nidulans* yielded 3 mg/l of active enzyme, while expression in *Escherichia coli* led to the formation of inclusion bodies and required time-consuming refolding of *Pe*AAO1 which was less stable than the native enzyme due to the lack of glycosylation (Ferreira et al. 2005; Ruiz-Dueñas et al. 2006). Aiming at enhanced expression in *Saccharomyces cerevisiae* and *Pichia pastoris*, *Pe*AAO1 was subjected to protein engineering using in vivo DNA shuffling and the mutagenic organized recombination process by homologous in vivo grouping (MORPHING) (Viña-Gonzalez et al. 2015, 2018). As result, two *Pe*AAO1 variants, FX7 and FX9, were constructed and expressed at concentrations of up to 25 mg/l. A recent review summarized different approaches of directed evolution to unlock *Pe*AAO1’s full potential for biotechnological purposes aiming at enhanced expression or acceptance of new substrates (Viña-Gonzalez and Alcalde 2020).

Recently, we cloned an aryl-alcohol oxidase *Pe*AAO2 from *P. eryngii* P34 in *P. pastoris* and produced it in a fed-batch process at a concentration of 315 mg/l (Jankowski et al. 2020). Interestingly, the protein sequences of *Pe*AAO2 and *Pe*AAO1 differ only in seven amino acid residues. Our efforts to actively express *Pe*AAO1 in *P. pastoris* failed and an explanation why the highly similar *Pe*AAO2 was expressed at high yields remained elusive. Here, we investigate the effect of the seven different amino acid residues on expression of *Pe*AAO1 in *P. pastoris.* A set of single, double and triple mutants of *Pe*AAO1 were generated and their expression levels were examined. The most active variants were produced in a fed-batch process, purified, and characterized. Homology models of the active variants were created in order to rationalize the effect of the mutations based on the structural changes. The gene copy numbers and mRNA levels of recombinant *P. pastoris* expressing *Pe*AAO1 variants were investigated by real-time PCR to determine the effects of these parameters on enzyme expression.

## Materials and methods

### Chemicals

All chemicals were of analytical grade or higher and purchased from Acros Organics (Geel, Belgium), AppliChem GmbH (Darmstadt, Germany), BD (Heidelberg, Germany), Carl Roth GmbH + Co. KG (Karlsruhe, Germany), J&K Scientific (Lommel, Belgium), and Sigma-Aldrich (Schnelldorf, Germany).

### Strains and plasmids

For all cloning procedures, chemically competent *Escherichia coli* DH5α cells were used (Clontech Laboratories Inc., Heidelberg, Germany). The expression of the *Pe*AAO1 variants was carried out using *P. pastoris* X-33 (recently reclassified as *Komagataella phaffii*) cells transformed with pPICZA-based plasmids containing the methanol inducible *AOX1*-promoter (Invitrogen, Carlsbad, USA).

### Site-directed mutagenesis

The gene *peaao1* encoding for *P. eryngii* ATCC 90787 aryl-alcohol oxidase 1 (GenBank accession number AF064069) was synthesized and cloned into pPICZA vector by BioCat GmbH (Heidelberg, Germany) in a codon optimized version (GenBank accession number MZ246833) for expression in yeast (JCat online tool) (Grote et al. 2005). The resulting plasmid pPICZA_*Pe*AAO1 was used as template for site-directed mutagenesis using the QuikChange protocol with the primers listed in Supplemental Table S1. First, seven single mutants, R152G, T265I, D361N, V367A, D512N, K583E, and Q584R, were generated according to the following procedure. One nanogram of pPICZA_*Pe*AAO1 was mixed with 500 nM each forward and reverse primer, 200 µM of each dNTP, 1 × high-fidelity buffer, 3% dimethyl sulfoxide (DMSO), and 0.02 U/µl Phusion High-Fidelity DNA-polymerase (Thermo Fisher Scientific, Bremen, Germany) in a total volume of 50 µl. Using a thermocycler, following cycling protocol was used: initial denaturation at 98 °C for 30 s, 16 times cycling of denaturation at 98 °C for 10 s, annealing for 30 s, extension at 72 °C for 75 s, followed by a final extension at 72 °C for 10 min, and a hold at 10 °C. The annealing temperature for each primer pair was calculated using the T_m_ calculator of Thermo Fisher Scientific.

To remove the parental plasmid, the reaction mixture was digested with FastDigest *Dpn*I (Thermo Fisher Scientific). Chemically competent *E. coli* DH5α cells were transformed with the digested sample and plated on selective LB agar plates (1% peptone, 0.5% yeast extract, 0.5% NaCl, 1.5% agar) containing 25 µg/ml Zeocin™ (InvivoGen, San Diego, USA). Up to five randomly selected colonies were used to inoculate 5 ml of LB medium with 25 µg/ml Zeocin™ and incubated overnight (37 °C, 180 rpm). Plasmid isolation was carried out using the ZR Plasmid Miniprep Kit (Zymo Research, Freiburg, Germany) following the manufacturer’s instructions. Introduction of mutations was verified through DNA sequencing by Eurofins Genomics Germany GmbH (Ebersberg, Germany). Once all seven single mutants were generated and evaluated regarding enzyme activity (see below), pPICZA_*Pe*AAO1_K583E was used as plasmid template to generate six double mutants (R152G/K583E, T265I/K583E, D361N/K583E, V367A/K583E, D512N/K583E, and K583E/Q584R) as described above. Finally, single mutations D361N and V367A were introduced in the double mutant K583E/Q584R (variant ER) using pPICZA_*Pe*AAO1_K583E/Q584R as template to construct the two triple mutants D361N/K583E/Q584R (variant NER) and V367A/K583E/Q584R (variant AER), respectively.

All generated pPICZA-based plasmids were linearized in the 5’*AOX1* region employing the FastDigest *Mss*I enzyme (Thermo Fisher Scientific). Electrocompetent *P. pastoris* X-33 cells were transformed with the linearized plasmids, plated on YPDS agar plates (1% yeast extract, 2% peptone, 2% dextrose, 1 M sorbitol, 2% agar) containing 100 µg/ml Zeocin™ and incubated at 30 °C until the formation of colonies.

### Enzyme production and purification

A number of *P. pastoris* transformants expressing either the mutants K583E/Q584R (variant ER), D361N/K583E/Q584R (variant NER) or V367A/K583E/Q584R (variant AER) were cultivated in 10 ml BMGY medium (1% yeast extract, 2% peptone, 100 mM potassium phosphate buffer pH 6, 1.34% yeast nitrogen base without amino acids, 4 × 10^−5^% biotin, 1% glycerol) in 100-ml shaking flasks overnight (30 °C, 200 rpm) and used to inoculate 10 ml of the expression medium BMMY (1% yeast extract, 2% peptone, 100 mM potassium phosphate buffer pH 6, 1.34% yeast nitrogen base without amino acids, 4 × 10^−5^% biotin and 0.5% (v/v) methanol) to an OD_600_ of 1. The expression was carried out for up to 48 h (25 °C, 200 rpm) and methanol was added every 24 h at 0.5% (v/v). OD_600_ value and volumetric activity were monitored daily and used to identify the best performing recombinant *Pichia* transformants.

Fed-batch cultivation of the selected *P. pastoris* transformants expressing ER, NER, and AER with glycerol as carbon source during the batch phase and 0.5% (v/v) methanol with 12 g/l *Pichia* trace metals (PTM_1_) solution during the fed-batch phase were carried out as described previously (Jankowski et al. 2020). Daily sampling was done to monitor cell growth, extracellular protein concentration, and volumetric activity. After 8 or 9 days of cultivation, the cells were harvested via centrifugation (11,325 × *g*, 15 min, 4 °C). The cell-free supernatant was concentrated and rebuffered using tangential flow filtration (TFF) and subsequently purified via three chromatographic steps, as described before for *Pe*AAO2 (Jankowski et al. 2020). In short, 5 ml of the first eluate from TFF was applied to a hydrophobic interaction chromatography column (Butyl Sepharose HP, GE Healthcare, Freiburg, Germany) and eluted with decreasing ammonium sulfate concentration. Active fractions were pooled, desalted, and loaded onto an anion exchange chromatography column (DEAE Sepharose FF, GE Healthcare) and eluted with increasing sodium chloride concentration. Again, active fractions were pooled and finally loaded onto a size exclusion chromatography column (Superdex 200 Increase 10/300 GL, GE Healthcare). The most active and purest fractions were concentrated, desalted, and stored at 4 °C until use. The production and chromatographic purification of *Pe*AAO2 were carried out as described before (Jankowski et al. 2020).

### Enzyme activity assay

The standard assay to assess AAO activity in the culture supernatant or of purified enzyme was carried out with veratryl alcohol as substrate at room temperature. 5 mM of veratryl alcohol was mixed with 100 mM sodium phosphate buffer pH 6, and the reaction was initiated with culture supernatant or an appropriate dilution of AAO containing sample in a 1-ml cuvette. The change of absorbance at 310 nm as a result of veratraldehyde formation $\left(\varepsilon_{310}={9,300\;\mathrm M}^{-1}\mathrm{cm}^{-1}\right)$ (Guillén et al. 1992) was followed using an Ultrospec 7000 photometer (GE Healthcare). Initial reaction rates were calculated according to Lambert–Beer law. Under the stated conditions, one unit of activity is defined as the amount of enzyme that converts 1 µmol substrate per minute.

### Protein quantification and *N*-deglycosylation

To determine protein concentrations at different steps of enzyme purification, the Bradford method was used employing bovine serum albumin as standard protein (Bradford 1976). The measurements were carried out at room temperature in 96-well micro titer plates using an Infinite M200 Pro plate reader (Tecan, Männedorf, Switzerland). The molar extinction coefficients of purified *Pe*AAO1 variants were calculated after heat denaturation of the samples and detection of released FAD as described for *Pe*AAO2 wild-type (Jankowski et al. 2020) and used for determination of molar enzyme concentrations. Latter ones were used to calculate enzyme concentration in fermentation supernatant, specific activities of purified enzymes, and kinetic constants.

*N*-Deglycosylation was performed using 20 µg of purified enzymes and peptide-*N*-amidase PNGase F (New England Biolabs, Frankfurt am Main, Germany) under denaturing conditions. For this, the samples were boiled at 100 °C for 10 min in the presence of SDS prior to deglycosylation with PNGase F. An aliquot of the deglycosylated samples as well as of the purified enzymes (each 5 µg) were loaded onto a 12.5% resolving gel and SDS-PAGE was conducted according to the protocol of Laemmli (1970).

### pH activity, stability, and melting temperature

The activity of *Pe*AAO1 variants ER, NER, and AER and *Pe*AAO2 wild-type towards the substrates *p*-anisyl alcohol and veratryl alcohol was determined in 100 mM Britton-Robinson buffer (consisting of 100 mM each boric acid, phosphoric acid, acetic acid) at different pH values ranging from pH 2 to 10 at room temperature. To determine the pH stability, the enzymes were incubated for up to 24 h in 100 mM Britton-Robinson buffer at pH 2 to 10 at room temperature. After certain time points, samples were taken and the relative activity towards veratryl alcohol was determined as described in the standard assay.

The melting temperature (*T*_M_) of the *Pe*AAO1 variants ER, NER, and AER was determined by the *Thermo*FAD assay (Forneris et al. 2009). The temperature at which 50% of the enzymatic activity is retained (*T*_50_) was determined for the *Pe*AAO1 variants as previously described (Jankowski et al. 2020).

### Specific activities

The activity of purified *Pe*AAO1 variants ER, NER, and AER as well as of *Pe*AAO2 was determined towards several AAO substrates at a final concentration of 5 mM in 100 mM sodium phosphate buffer pH 6 at room temperature. The assay was performed in 96-well UV-Star® micro titer plates (Greiner Bio-One GmbH, Frickenhausen, Germany). The conversion of the selected alcohols to their corresponding aldehydes was followed spectrophotometrically using the Infinite M200 Pro plate reader (Tecan): $p-\mathrm{anisyl}\;\mathrm{alcohol}\;\left(\varepsilon_{285}={16,980\;\mathrm M}^{-1}\mathrm{cm}^{-1}\right)$ (Guillén et al. 1992), $\mathrm{benzyl}\;\mathrm{alcohol}\;\left(\varepsilon_{250}={13,800\;\mathrm M}^{-1}\mathrm{cm}^{-1}\right)$ (Guillén et al. 1992), $\mathrm{piperonyl}\;\mathrm{alcohol}\;\left(\varepsilon_{317}={8,680\;\mathrm M}^{-1}\mathrm{cm}^{-1}\right)$ (Jankowski et al. 2020), $\mathrm{veratryl}\;\mathrm{alcohol}\;\left(\varepsilon_{310}={9,300\;\mathrm M}^{-1}\mathrm{cm}^{-1}\right)$ (Guillén et al. 1992), and *trans,trans*-2,4-hexadienol $\left(\varepsilon_{280}={30,140\;\mathrm M}^{-1}\mathrm{cm}^{-1}\right)$ (Ruiz-Dueñas et al. 2006).

### Determination of kinetic parameters

The kinetic parameters of the oxidation of *p*-anisyl alcohol (0.98 to 1,000 µM) and veratryl alcohol (9.8 to 10,000 µM) with the purified *Pe*AAO1 variants ER, NER, and AER were determined in 96-well UV-Star® micro titer plates at 25 °C using an Infinite M200 Pro plate reader (Tecan). An appropriate stock solution of purified enzyme was mixed with 100 mM sodium phosphate buffer pH 6 and substrate stocks with varying concentrations. Using the program OriginPro 2019 (OriginLab Corporation, Northampton, MA, USA), a non-linear fit according to the Michaelis–Menten equation was calculated and the parameters V_max_ and K_M_ were extracted and used for calculation of k_cat_ and k_cat_/K_M_ values.

### Real-time PCR to determine gene copy numbers and mRNA levels

For extraction of genomic DNA (gDNA) in order to determine the gene copy numbers, precultures of recombinant *P. pastoris* transformants expressing different *Pe*AAO1 variants were cultivated in 10 ml BMGY medium overnight (30 °C, 200 rpm). The gDNA was extracted using the *Quick*-DNA Fungal/Bacterial Miniprep Kit (Zymo Research, Freiburg, Germany) according to the manufacturer’s protocol and eluted in 50 µl of ultra-pure water. The precultures were used to inoculate 10 ml of BMM medium (100 mM potassium phosphate buffer pH 6, 1.34% yeast nitrogen base without amino acids, 4 × 10^−5^% biotin, and 0.5% (v/v) methanol) to an OD_600_ of 1, and cultures were incubated for 48 h (25 °C, 200 rpm). Samples diluted to an OD_600_ of 1 were used for total RNA extraction using the RNeasy Mini Kit (Qiagen, Hilden, Germany) according to the manufacturer’s protocol. On-column digestion of residual DNA with RNase-free DNase set (Qiagen) during purification was implemented. gDNA and RNA concentrations were measured spectrophotometrically using the NanoQuant™ Plate with the Infinite M200 Pro plate reader (Tecan) by determining the 260 nm absorbance and 260/280 nm ratio. One hundred nanograms of total RNA was used for cDNA synthesis using SuperScript™ III Reverse Transcriptase (Invitrogen) with 2.5 µM Oligo(dT)_18_ primers (Thermo Fisher Scientific) as described in the manufacturer’s protocol. The resulting cDNA was diluted 1:4 with ultra-pure water.

Real-time-PCR reactions were set up with innuMIX qPCR DSGreen Standard Mix (AnalytikJena, Jena, Germany) according to the manufacturer’s protocol and conducted on real-time PCR cycler qTOWER^3^ *touch* (AnalytikJena). Either 2 ng of gDNA or 2 µl of diluted cDNA sample was used in triplicate. The cycling protocol was as followed: initial denaturation at 95 °C for 120 s, followed by 40 cycles of denaturation at 95 °C for 30 s, and combined annealing and detection at 60 °C for 45 s. A melting curve analysis was included after the PCR run from 60 to 95 °C in 0.5 °C increments.

For amplification of target *peaao* genes, specific primers 1qPCR_fw (5’-3’: TCCAGTTGCTAGAGGTGACATC) and 1qPCR_rev (5’-3’: TGGGTCGAATGGTCTGATAACG) were used, while actin was used as reference gene with primers Actin_fw (5’-3’: GGTATTGCTGAGCGTATGCAAA) and Actin_rev (5’-3’: CCACCGATCCATACGGAGTACT). Optimal primer concentrations were determined by titrating forward and reverse primers at 100 to 300 nM each, and combinations yielding no amplification in the no-template control were used. Primer efficiencies for both pairs at optimal concentrations were calculated using dilution series of gDNA. The results of gene copy number and mRNA level determination were analyzed using the software qPCRsoft 4.1 (AnalytikJena) employing the primer efficiency corrected Pfaffl method and actin as reference gene (Pfaffl 2001).

### Homology modelling

The crystal structure of *Pe*AAO1 wild-type (PDB entry 3FIM) (Fernández et al. 2009) was used as template to generate homology models of *Pe*AAO1 variants ER, NER, and AER using the online tool SWISS-MODELL (Waterhouse et al. 2018) and the program PyMOL for visualization.

## Results

### Effect of mutations on *Pe*AAO1 expression

**Fig. 1:**
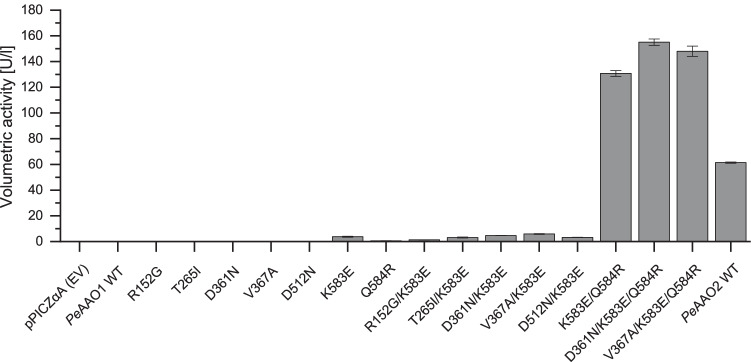
Volumetric activities [U/l] of *Pe*AAO2 wild-type and *Pe*AAO1 variants in the supernatant of small-scale expressions of recombinant *P. pastoris* towards veratryl alcohol (5 mM). Empty vector pPICZαA (EV) was used as negative control. Activities were measured after 48 h of cultivation in BMMY medium (25 °C and 200 rpm) with 0.5% (v/v) methanol added daily

To study the effect of the seven amino acid residues which differ in the highly expressed *Pe*AAO2 and *Pe*AAO1, not expressed in *P. pastoris*, at first, seven single mutants of *Pe*AAO1 with the substitutions R152G, T265I, D361N, V367A, D512N, K583E, and Q584R were created. The corresponding *P. pastoris* transformants were screened for AAO activity after expression in BMMY medium in shaking flasks. Both AAOs, *Pe*AAO1, and *Pe*AAO2 have been reported to catalyze the oxidation of veratryl alcohol to veratraldehyde (Guillén et al. 1992; Jankowski et al. 2020). Thus, conversion of veratryl alcohol by samples taken from the supernatant after expression and cell centrifugation was used for expression verification. Out of the seven single mutants, activity was only detectable for the K583E variant and reached 3.7 U/l (Fig. 1).

Despite the low volumetric activity of *Pe*AAO1 variant K583E compared to *Pe*AAO2 wild-type with 61 U/l, the successful expression of this variant in *P. pastoris* was a good starting point for further mutagenesis. In the next step, six *Pe*AAO1 double mutants were created by introducing mutations R152G, T265I, D361N, V367A, D512N, and Q584R, respectively, into the *Pe*AAO1 variant K583E and screened for improved volumetric activity towards veratryl alcohol. Remarkably, the double mutant K583E/Q584R reached a volumetric activity of up to 131 U/l (Fig. 1), which is roughly 35 times higher than that of the variant K583E and even surpasses the volumetric activity of *Pe*AAO2 wild-type by factor 2. The double mutants D361N/K583E and V367A/K583E showed slightly increased activities (up to 4.7 U/l and 5.9 U/l, respectively) compared to the starting variant K583E (3.7 U/l), indicating a beneficial effect of the mutations D361N and V367A on expression and/or enzyme activity. Mutations D361N and V367A, respectively, were introduced into the best double mutant K583E/Q584R yielding two triple mutants. An alignment of *Pe*AAO1 and *Pe*AAO2 wild-type and of *Pe*AAO1 variants is given in Supplemental Fig. S1. After expression in *P. pastoris*, a volumetric activity of up to 155 U/l for the variant *Pe*AAO1 D361N/K583E/Q584R was achieved (Fig. 1), which is 1.2-fold higher than the parental double mutant K583E/Q584R and 2.5-fold higher than *Pe*AAO2 wild-type. The *Pe*AAO1 mutant V367A/K583E/Q584R was expressed in *P. pastoris* leading to a similar volumetric activity of 148 U/l. The *Pe*AAO1 variants K583E/Q584R (ER), D361N/K583E/Q584R (NER), and V367A/K583E/Q584R (AER) were selected for production at a larger scale and characterization.

### Production in a bioreactor and purification of active *Pe*AAO1 variants

The fed-batch cultivation of recombinant *P. pastoris* to produce *Pe*AAO1 variants ER, NER, and AER was conducted for 8 to 9 days. *Pe*AAO1 variant ER was produced at a volumetric activity of 4898 U/l after 160 h (Table 1). At around the same time, NER reached 5263 U/l and AER yielded 5734 U/l. All enzymes were purified to homogeneity following the established three-step purification protocol (see the experimental section). Purified *Pe*AAO1 NER exhibited the highest specific activity towards veratryl alcohol with 48.4 U/mg, followed by AER with 45.6 U/mg. *Pe*AAO1 ER showed a slightly lower specific activity with 42.4 U/mg similar to *Pe*AAO2 wild-type with 43 U/mg. Molar protein concentrations calculated after purification revealed that the *Pe*AAO1 variant ER was produced at a level of 116 mg/l, followed by 113 mg/l for NER and 98 mg/l for AER.

**Table 1:** Activity and enzyme production during fed-batch cultivation and properties of the purified *Pe*AAO1 variants and *Pe*AAO2 wild-type

Enzyme	*Pe*AAO1 ER	*Pe*AAO1 NER	*Pe*AAO1 AER	*Pe*AAO2 WT
Volumetric activity after ~ 160 h [U/l]^a^	4898 (160 h)	5263 (159 h)	5734 (160 h)	5229 (167 h)
Enzyme concentration [mg/l]^b^	116 (160 h)	113 (183 h)	98 (179 h)	169 (214 h)
Specific activity [U/mg]^c^	42.4	48.4	45.6	43.0
Absorbance maxima [nm]	463, 383	463, 383	463, 383	463, 376 ^d^
ε_463_ extinction coefficient [M^−1^ cm^−1^]	8687	8608	8432	7029 ^d^

^a^For comparison, volumetric activities after roughly 160 h (time of harvest for *Pe*AAO1 ER) are shown. Enzymatic activity was determined with 5 mM veratryl alcohol in 100 mM sodium phosphate buffer pH 6

^b^Enzyme concentration calculated based on molar protein concentration of purified enzyme. Time of harvest is given in parenthesis

^c^Specific activity of purified enzyme towards veratryl alcohol based on molar protein concentrations

^d^Values from (Jankowski et al. 2020)

To elucidate the reasons for different expression yields of the *Pe*AAO1 variants, their properties were investigated. All *Pe*AAO1 mutants exhibited absorption spectra typical for flavoproteins with two absorbance maxima at 463 nm and 383 nm. The molar extinction coefficients were calculated based on absorbance of the released FAD after heat precipitation of the apoprotein and ranged between 8400 and 8700 M^−1^ cm^−1^ (Table 1).

**Fig. 2:**
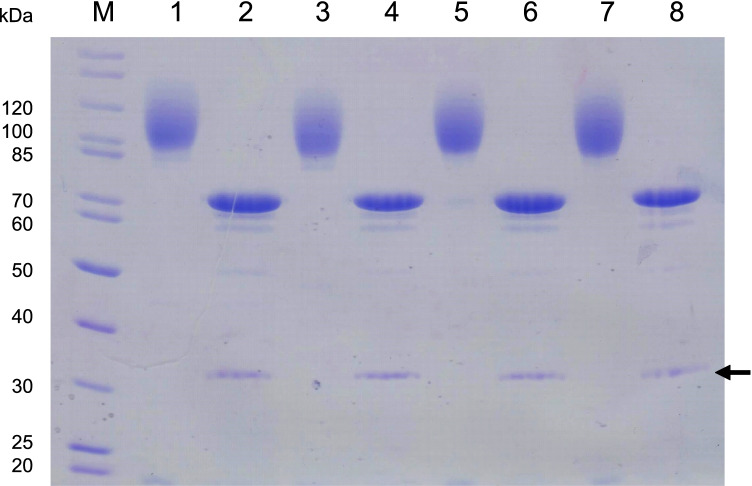
SDS-PAGE analysis of purified and *N*-deglycosylated *Pe*AAO2 wild-type and *Pe*AAO1 variants. M, PageRuler™ marker; 1, native *Pe*AAO2; 2, deglycosylated *Pe*AAO2; 3, native *Pe*AAO1 ER; 4, deglycosylated *Pe*AAO1 ER; 5, native *Pe*AAO1 NER; 6, deglycosylated *Pe*AAO1 NER; 7, native *Pe*AAO1 AER; 8, deglycosylated *Pe*AAO1 AER. Arrow indicates PNGase F (36 kDa). 5 µg of each sample was loaded and the gel was stained with Coomassie Brilliant Blue R250

Purified *Pe*AAO2 wild-type and *Pe*AAO1 variants exhibited similar apparent molecular masses of around 100 kDa according to SDS-PAGE (lanes 1, 3, 5, and 7 in Fig. 2), while the theoretical molecular weight (without the predicted signal peptide) was 61 kDa for *Pe*AAO2 and *Pe*AAO1 variants. After *N*-deglycosylation sharp bands at 70 kDa appeared for all enzymes (lanes 2, 4, 6, and 8 in Fig. 2), indicating 30% of *N*-glycosylation of recombinantly produced *Pe*AAO1 variants and *Pe*AAO2 wild-type.

### pH activity and stability

Regarding pH optimum, activity of *Pe*AAO2 wild-type and *Pe*AAO1 variants towards *p*-anisyl alcohol and veratryl alcohol was highest at slightly acidic pH values. The highest activity towards *p*-anisyl alcohol was at pH 5 for *Pe*AAO2 wild-type and *Pe*AAO1 NER, while pH 6 was best for *Pe*AAO1 ER and AER (Supplemental Fig. S2a). *Pe*AAO2 wild-type and all *Pe*AAO1 variants showed the highest activity at pH 6 with veratryl alcohol (Supplemental Fig. S2b).

**Fig. 3:**
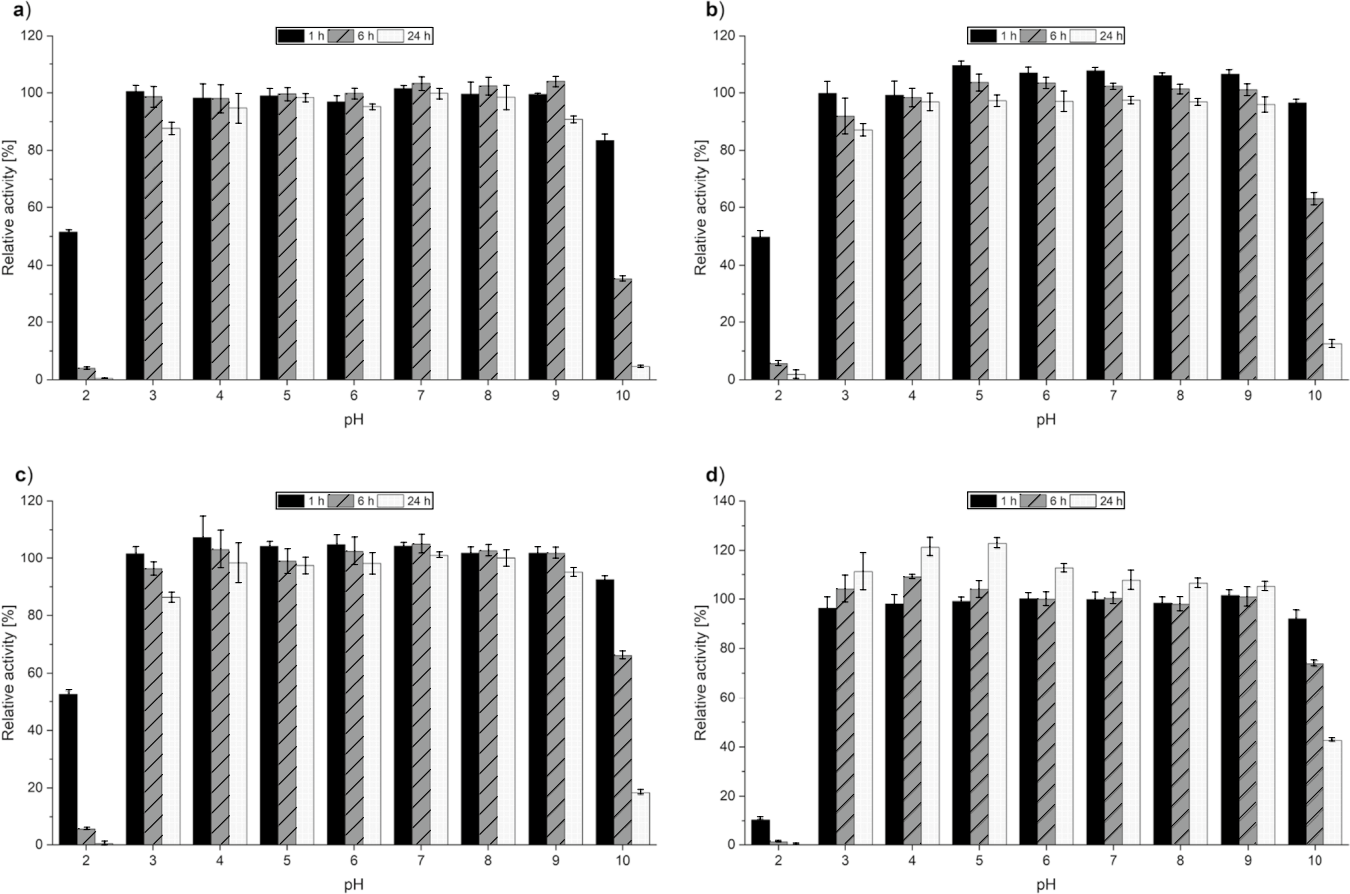
pH stability profile of *Pe*AAO1 variants **a** ER, **b** NER, **c** AER, and **d** *Pe*AAO2 wild-type after incubation in 100 mM Britton-Robinson buffer pH 2 to 10 for up to 24 h. After 1 h: black, filled; after 6 h: gray, striped; after 24 h: white, dotted. Initial activity without incubation was set to 100%

High pH stability was found for all three *Pe*AAO1 variants ER, NER and AER with relative activities of over 85% after 24 h of incubation at pH ranging from 3 to 9 (Fig. 3a, b, and c). After 24 h incubation at pH 10, activities dropped, and completely vanished after incubation at pH 2. *Pe*AAO2 wild-type showed up to 130% increased relative activities after 24 h incubation between pH 3 and 6 (Fig. 3d). Incubation at pH 2 for 1 h seemed to have a more adverse effect on *Pe*AAO2 wild-type as on the *Pe*AAO1 variants, while *Pe*AAO2 wild-type remained more active after incubation at pH 10 with 40% relative activity as compared to the *Pe*AAO1 variants.

### Thermal stability

The melting temperature *T*_M_ was determined by measuring the FAD fluorescence during unfolding of the proteins at increasing temperatures. The *T*_M_ values for all *Pe*AAO1 variants as well as for *Pe*AAO2 wild-type were at around 65 °C (Table 2), while the *T*_50_ value at which half of the enzymatic activity lost after 10 min incubation was roughly 3 to 4 °C lower for all enzymes at around 61.5 °C.

**Table 2:** Melting temperature (*T*_M_) and T*T*_50_ value of *Pe*AAO1 variants and *Pe*AAO2 wild-type

Enzyme	*Pe*AAO1 ER	*Pe*AAO1 NER	*Pe*AAO1 AER	*Pe*AAO2 WT ^a^
*T*_M_ [°C]	65.5	65.0	64.5	65.5
*T*_50_ [°C]	61.5	61.6	61.7	62.1

^a^Values from (Jankowski et al. 2020)

### Specific activities and kinetic parameters

Next, the specific activity of *Pe*AAO1 variants towards *p*-anisyl alcohol, benzyl alcohol, *trans*,*trans*-2,4-hexadienol, piperonyl alcohol, and veratryl alcohol was determined. In general, specific activities of the *Pe*AAO1 variants and *Pe*AAO2 wild-type were in the same range when using the same substrate (Supplemental Fig. S3). The highest specific activity was reached with *trans*,*trans*-2,4-hexadienol of up to 89 U/mg for AER, followed by *p*-anisyl alcohol with up to 67 U/mg (NER). The lowest specific activities of 13 U/mg or below were observed during the oxidation of benzyl alcohol.

Kinetic measurements for the oxidation of *p*-anisyl alcohol and veratryl alcohol catalyzed by *Pe*AAO1 variants were conducted at pH 6. The *Pe*AAO1 variant NER showed the highest k_cat_ values with 87.9 s^−1^ and 68.2 s^−1^ for *p*-anisyl alcohol and veratryl alcohol, respectively, and also reached the highest catalytic efficiencies with 1975 mM^−1^ s^−1^ and 124.7 mM^−1^ s^−1^, respectively, among the three *Pe*AAO1 variants (Table 3). The K_M_ values for *p*-anisyl alcohol for the *Pe*AAO1 variants were in the range from 39.2 µM for ER to 48.8 µM for AER. The K_M_ values for veratryl alcohol were roughly 10 times higher than for *p*-anisyl alcohol and ranged from 541.9 µM for AER to 549.0 µM for ER. *Pe*AAO2 wild-type showed the lowest K_M_ values with 24.3 µM and 446.6 µM for *p*-anisyl alcohol and veratryl alcohol, respectively, and in case of *p*-anisyl alcohol also the highest overall catalytic efficiency with 2436 mM^−1^ s^−1^.

**Table 3:** Kinetic parameters of *Pe*AAO1 variants and *Pe*AAO2 wild-type towards the substrates *p*-anisyl alcohol and veratryl alcohol in 100 mM sodium phosphate buffer pH 6 at 25 °C

Substrate	Enzyme	K_M_ [µM]	k_cat_ [s^−1^]	k_cat_/K_M_ [mM^−1^ s^−1^]
*p*-Anisyl alcohol	*Pe*AAO1 ER	39.2 ± 1.9	73.8 ± 0.03	1883
	*Pe*AAO1 NER	44.5 ± 1.5	87.9 ± 0.06	1975
	*Pe*AAO1 AER	48.8 ± 1.5	78.2 ± 0.05	1602
	*Pe*AAO2 WT ^a^	24.3	59.2	2436
Veratryl alcohol	*Pe*AAO1 ER	549.0 ± 12.6	54.9 ± 0.02	100.0
	*Pe*AAO1 NER	546.7 ± 5.8	68.2 ± 0.05	124.7
	*Pe*AAO1 AER	541.9 ± 6.4	58.5 ± 0.03	108.0
	*Pe*AAO2 WT ^a^	446.6	47.2	105.7

^a^Values from (Jankowski et al. 2020)

### Gene copy number and mRNA levels

**Fig. 4:**
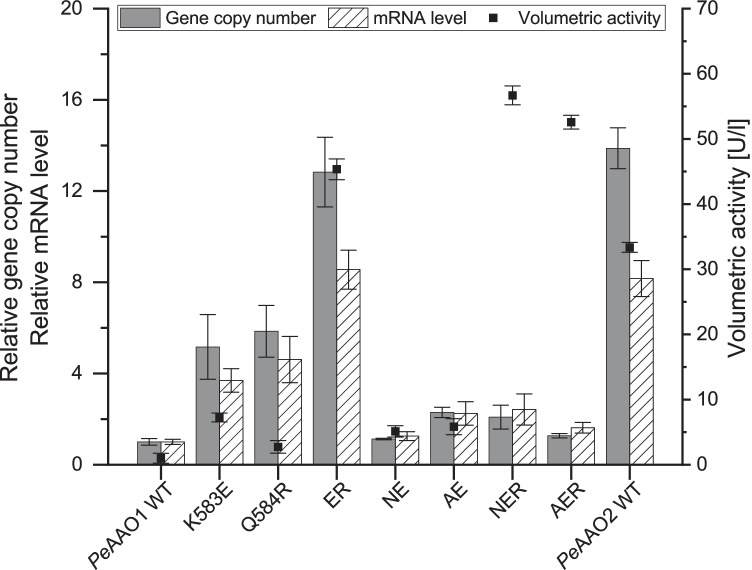
Relative gene copy number and mRNA level determination in correlation to volumetric activity [U/l] of *Pe*AAO1 variants and *Pe*AAO2 wild-type. Actin was used as reference gene and values are depicted as ratio of target gene and reference gene. Volumetric activity after 48 h expression in BMM medium (25 °C, 200 rpm) was determined towards 5 mM veratryl alcohol in 100 mM sodium phosphate buffer pH 6. Relative gene copy number: gray column, filled; relative mRNA level: white column, striped; volumetric activity: black squares. ER: double mutant K583E/Q584R; NE: double mutant D361N/K583E; AE: double mutant V367A/K583E; NER: triple mutant D361N/K583E/Q584R; AER: triple mutant V367A/K583E/Q584R

Gene copy number and mRNA level of the target *peaao* genes of the most active *P. pastoris* transformant each expressing *Pe*AAO1 wild-type, *Pe*AAO1 variants K583E, Q584R, K583E/Q584R (ER), D361N/K583E (NE), V367A/K583E (AE), NER, AER, and *Pe*AAO2 wild-type, respectively, were determined (Fig. 4). The number of integrated genes varied between one for *Pe*AAO1 wild-type and roughly 14 for *Pe*AAO2 wild-type. The mRNA level increased with increasing gene copy number. However, although the volumetric activity was highest for *Pe*AAO1 variants NER and AER with 56 and 52 U/l, respectively, the detected gene copy numbers and mRNA levels were roughly between one and two and thus among the lowest detected values. Interestingly, the *P. pastoris* transformant expressing *Pe*AAO1 variant ER contained roughly 13 gene copies but showed a similar volumetric activity of 45 U/l as NER and AER with one to two gene copies and 3–4 times lower mRNA levels.

## Discussion

The sequence of *Pe*AAO2 was mimicked by mutating *Pe*AAO1 in a successive manner. Out of seven single *Pe*AAO1 mutants, only the mutation K583E led to measurable activity towards veratryl alcohol after expression in *P. pastoris*. By combining the mutations K583E and Q584R, volumetric activity increased from 3.7 to 131 U/l, even though no functional expression was achieved for the single mutant Q584R. Here, we observe a synergistic positive effect of these two mutations on heterologous expression. The introduction of either the mutation D361N or V367A further increased volumetric activities of the respective *Pe*AAO1 variants NER and AER to 155 and 148 U/l.

**Fig. 5:**
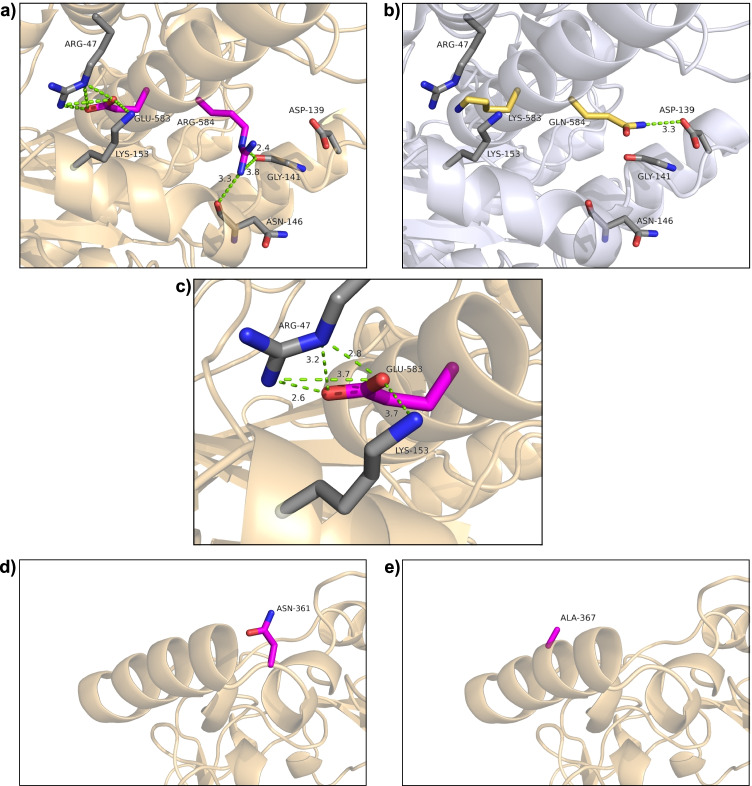
Location of mutations K583E/Q584R in **a** *Pe*AAO1 variant ER and **b** the same positions in wild-type *Pe*AAO1; **c** close-up on possible polar contacts of variant ER; **d** mutation D361N in *Pe*AAO1 NER and **e** mutation V367A in *Pe*AAO1 AER. The two introduced mutations leading to Glu583 and Arg584 are depicted in pink; amino acid residues and backbone atoms in close proximity to form polar contacts (Arg47, Lys153, Asn146, Gly141, Asp139) are depicted in gray **(a)**. View of the same positions in wild-type *Pe*AAO1 with Lys583 and Gln584 depicted in yellow **(b)**. Close-up on possible polar contacts in variant ER at position 583 **(c)**. The introduced mutations D361N and V367A in variants NER and AER are depicted in pink **(d** and **e)**. The homology models of *Pe*AAO1 variants ER, NER, and AER were created using the crystal structure of *P. eryngii* AAO (wild-type *Pe*AAO1) (PDB entry 3FIM) as template. The possible polar contacts are colored in green and predicted distances are given in Å

In an attempt to rationalize the positive effect of the four mutations on expression in *P. pastoris*, the *Pe*AAO1 variants were investigated at different levels. First, homology models constructed for the *Pe*AAO1 variants revealed that the neighboring mutations K583E and Q584R in the variant ER are located in the C-terminal α-helix close to the surface of the enzyme (Fig. 5a). At position 583, the positively charged lysine in *Pe*AAO1 wild-type (Lys583 in Fig. 5b) was replaced by the negatively charged glutamate (Glu583 in Fig. 5a), while at position 584, a polar glutamine (Gln584) was substituted with a positively charged arginine (Arg584). The introduction of Glu583 enables polar interactions with Arg47 and Lys153, both located less than 4 Å away from Glu583 (Fig. 5c). Barlow and Thornton investigated the distance distribution of ion pairs in 38 structures of proteins and defined the distance of ≤ 4 Å between two charged residues as criterion to form an ion pair (Barlow and Thornton 1983). It was shown that close-range electrostatic interactions between charged amino acid residues as in salt bridges contribute, among others, to protein folding and stability (Kumar and Nussinov 2002).

The positive charge of the guanidine moiety in Arg47 is delocalized among the three nitrogen atoms, which increases the probability to form ion pairs with suitable oppositely charged residues like Glu583 (Barlow and Thornton 1983). The ε-amino group of Lys153 is also only 3.7 Å away from Glu583 and thus can also be involved in salt bridge formation. Musafia et al. performed an extended structure-based analysis of simple and complex salt bridges in 94 proteins and concluded that a central negatively charged glutamate residue can interact with an arginine via one or two bonds and additionally with a lysine via one bond, given that all charged groups are within the appropriate distance (Musafia et al. 1995). Thus, the mutation K583E might be involved in a new complex salt bridge formation with Arg47 and Lys153 in the C-terminal α-helix of the enzyme and might thereby influence the expression of *Pe*AAO1 in *P. pastoris* by enhancing protein folding. *Pe*AAO1 variant ER containing the additional mutation Q584R showed a dramatic enhancement of volumetric activity in comparison to K583E by factor 35, although the mutation Q584R alone did not lead to any observable expression. The homology model indicates that Arg584 is not in the ideal proximity to form salt bridges with other charged residues but might establish hydrogen bonds with main chain carbonyl groups of Asn146 and/or Gly141 with distances between 2.4 and 3.8 Å, respectively (Fig. 5a). It has been proposed that arginine is able to participate in several hydrogen bonds with main chain carbonyl oxygens and thereby connect different structural elements, enhancing protein stability as well (Borders et al. 1994). Apparently both mutations K583E and Q584R have a synergistic effect on expression, probably due to improved folding or protein stability during processing which might positively influence protein translocation, FAD incorporation, or protein glycosylation. Possibly, once the mutation Q584R is introduced to generate variant ER, the protein folding is slightly altered and the proposed salt bridges of Glu583 can be formed which eventually leads to the increased expression of this enzyme variant. It is important to point out that all assumptions are based on homology models built on the basis of the crystal structure of the non-glycosylated holoprotein *Pe*AAO1, crystallized after expression and refolding from inclusion bodies in *E. coli* (Fernández et al. 2009). Glycan moieties present in the three *Pe*AAO1 variants might influence folding of the enzymes so that crystal structures might differ from the predicted ones. Moreover, the mutations introduced in *Pe*AAO1 could have an impact on FAD binding as the position Arg47 involved in the hypothesized salt bridge is located in an N-terminal helix closely located to the highly conserved dinucleotide-binding motif (Fernández et al. 2009). Crystal structures of the glycosylated *Pe*AAO2 and the *Pe*AAO1 variants expressed in *P. pastoris* could provide more insight into possible structural changes induced by the introduced mutations. However, due to the heterogeneity caused by attached glycan moieties, crystallization of glycoproteins is a challenging task (Chang et al. 2007) and up to now has only been described for one glycosylated AAO -* Mt*AAOx from *Thermothelomyces thermophilus* (Kadowaki et al. 2020).

The additionally introduced mutations D361N or V367A are located in a surface exposed α-helix on the enzyme surface (Fig. 5d and e), which is unique for AAOs in comparison to other family members of the glucose-methanol-choline (GMC) oxidoreductase superfamily (Fernández et al. 2009). While no direct conclusion for the effect of the mutation V367A can be drawn based on structure-related changes, the mutation D361N introduces a new potential *N*-glycosylation site with the general motif of Asn-X-Ser/Thr, where X is any amino acid except for proline (Kukuruzinska et al. 1987). Glycosylation may assist various processes like protein folding (Helenius and Aebi 2004), stabilization of mature protein structures (Imperiali and O'Connor 1999; Wormald and Dwek 1999; Wyss and Wagner 1996), and thermostability of the protein (Wang et al. 1996). With the newly introduced *N*-glycosylation site, *Pe*AAO1 variant NER contains a total of eight potential sites which are identical to *Pe*AAO2 wild-type (Jankowski et al. 2020). All three *Pe*AAO1 variants and *Pe*AAO2 wild-type exhibit highly similar molecular weights and *N*-glycosylation contents according to SDS-PAGE and PNGase F treatment as well as similar high pH and thermostability. The high pH stability of all variants is comparable to that observed for *Pe*AAO1 variant FX7 expressed in *S. cerevisiae*, which contained 50% glycosylation content (Viña-Gonzalez et al. 2015). It should be noted that most likely not all predicted *N*-glycosylation sites are in fact glycosylated. A database-survey focused on glycoproteins revealed that on average only two thirds of *N*-glycosylation sites are occupied (Apweiler et al. 1999). For example, the *Mt*AAOx from *T. thermophilus* contains six predicted *N*-glycosylation sites and X-ray structure elucidation revealed that four of them were glycosylated while one of them presents the major *N*-glycosylation site (Kadowaki et al. 2020). Interestingly, the previously reported optimized FX9 variant of *Pe*AAO1 expressed in *P. pastoris* was poorly glycosylated despite the presence of seven predicted *N*-glycosylation sites (Viña-Gonzalez et al. 2018), identical to the ones present in *Pe*AAO1 ER and AER. Maybe Asn361 in NER is only slightly *N*-glycosylated and therefore only marginal differences in glycan pattern of NER and the other variants exist that are not detectable via SDS-PAGE. In this case, the additional *N*-glycosylation in variant NER (and NE) could positively affect protein folding and/or secretion of the enzyme leading to enhanced volumetric activities. Stabilities of the purified *Pe*AAO1 variants and *Pe*AAO2 wild-type were comparable and showed no major differences: all *Pe*AAO1 variants exhibited high pH stabilities with roughly 90% of activity after incubation at pH 3 to 9 for 24 h, *T*_M_ values at around 65 °C, and *T*_50_ values between 61.5 and 62 °C.

The gene copy numbers and mRNA levels varied among the recombinant *P. pastoris* strains. Most strikingly, strains expressing the variants AER and NER demonstrated only one to two gene copies and similar high mRNA levels while exhibiting the highest observed volumetric activities. By comparing these data with the strain expressing the double mutant ER, which contained roughly 13 copies and showed a slightly lower volumetric activity than the triple mutants, our results indicate that the same (or even higher) level of volumetric AAO activity can be achieved with the triple mutants likely due to lower metabolic burden with just one to two gene copies. Overall, the real-time PCR results indicate that the observed higher volumetric activity of variants NER and AER can be solely attributed to effects of the mutations rather than multiple integration of the *aao* genes.

Since volumetric activity is dependent on enzyme concentration, on the one hand, and on catalytic properties of this enzyme, on the other hand, it is important to compare the catalytic parameters of the mutants. In general, catalytic activity of all *Pe*AAO1 variants was higher compared to *Pe*AAO2 wild-type. Thereby, mutations D361N and V367A seem to have a stimulating effect on catalytic activity when combined with ER mutations. The specific activities of the *Pe*AAO1 variants NER and AER measured for several substrates were slightly higher than those of *Pe*AAO1 ER and even *Pe*AAO2 wild-type. The α-helix which harbors both positions is the most external structural element in proximity to the catalytic pocket and might therefore influence substrate access to the active site. Interestingly, *Pe*AAO2 wild-type showed lowest K_M_ values for *p*-anisyl alcohol and veratryl alcohol (Jankowski et al. 2020). *Pe*AAO1 variant NER showed the highest k_cat_ value among all *Pe*AAO1 variants and *Pe*AAO2 wild-type, followed by variant AER, which could explain, at least to some extent, their increased volumetric activities after expression compared to *Pe*AAO2.

Comparison of catalytic properties of the *Pe*AAO1 variants with other AAOs showed that catalytic efficiencies of the *Pe*AAO1 variants are in the same range as compared to the poorly glycosylated *Pe*AAO1 variant FX9 expressed in *P. pastoris* with efficiencies of 1909 mM^−1^ s^−1^ (*p*-anisyl alcohol) and 139 mM^−1^ s^−1^ (veratryl alcohol) (Viña-Gonzalez et al. 2018). Interestingly, *Pe*AAO1 wild-type expressed in *A. nidulans* showed higher catalytic efficiencies with 5233 mM^−1^ s^−1^ (*p*-anisyl alcohol) and 210 mM^−1^ s^−1^ (veratryl alcohol) and up to two times higher k_cat_ values with 142 s^−1^ and 114 s^−1^ for *p*-anisyl alcohol and veratryl alcohol, respectively (Ferreira et al. 2006). While *Um*AAO from *Ustilago maydis* expressed in *P. pastoris* exhibited higher catalytic efficiencies towards *p*-anisyl alcohol (9380 mM^−1^ s^−1^) and veratryl alcohol (440 mM^−1^ s^−1^) (Couturier et al. 2016), than the *Pe*AAO1 variants, r*Cc*AAO from *Coprinopsis cinerea* expressed in *P. pastoris* showed a lower catalytic efficiency for conversion of *p*-anisyl alcohol with 1077 mM^−1^ s^−1^ (Tamaru et al. 2018). On the other hand, r*Cc*AAO showed an up to two times higher catalytic efficiency towards veratryl alcohol and a higher substrate affinity with K_M_ of 48.3 µM as compared to 446 to 549 µM for the *Pe*AAO1 variants. *Mt*AAOx from *T. thermophilus* showed quite low catalytic efficiencies with only 0.007 mM^−1^ s^−1^ and 0.011 mM^−1^ s^−1^ towards *p*-anisyl alcohol and veratryl alcohol, respectively (Kadowaki et al. 2020).

In conclusion, site-directed mutagenesis of *P. eryngii Pe*AAO1 led to the generation of three active and readily expressible enzyme variants with expression levels exceeding those of the already described *Pe*AAO1 variants. Up to now, the highest expression reported for the FX9 variant of *Pe*AAO1 in *P. pastoris* was 25.5 mg/l (Viña-Gonzalez et al. 2018). Here, *Pe*AAO1 variants ER, NER, and AER were constructed and expressed at four to fivefold higher concentrations ranging between 98 and 116 mg/l, accompanied by high volumetric activities. All enzymes could be produced in a bioreactor at 3 l scale, purified, and characterized.

The synergistic stabilizing effect caused by the introduced mutations K583E and Q584R is hypothesized. The introduced mutations also slightly affected the catalytic properties of the enzyme variants. In future studies, the beneficial effect of mutations K583E/Q584R on *Pe*AAO1 expression could be combined with the reported mutations affecting enzyme selectivity and activity, such as in the oxidation of secondary benzylic alcohols (Viña-Gonzalez et al. 2019). This way, enzyme variants with new or improved catalytic activities and enhanced expression yields could become easily accessible for large-scale biocatalytic applications.

## Supplementary Information

Below is the link to the electronic supplementary material.

Supplementary file1 (PDF 234 KB)

## Data Availability

All data on which the conclusions were drawn are presented in this study.

## References

[CR1] Apweiler R, Hermjakob H, Sharon N (1999). On the frequency of protein glycosylation, as deduced from analysis of the SWISS-PROT database. Biochim Biophys Acta.

[CR2] Barlow DJ, Thornton JM (1983). Ion-pairs in proteins. J Mol Biol.

[CR3] Borders CL, Broadwater JA, Bekeny PA, Salmon JE, Lee AS, Eldridge AM, Pett VB (1994). A structural role for arginine in proteins: multiple hydrogen bonds to backbone carbonyl oxygens. Protein Sci.

[CR4] Bradford MM (1976). A rapid and sensitive method for the quantitation of microgram quantities of protein utilizing the principle of protein-dye binding. Anal Biochem.

[CR5] Carro J, Ferreira P, Martínez ÁT, Gadda G (2018). Stepwise hydrogen atom and proton transfers in dioxygen reduction by aryl-alcohol oxidase. Biochemistry.

[CR6] Carro J, Ferreira P, Rodríguez L, Prieto A, Serrano A, Balcells B, Ardá A, Jiménez-Barbero J, Gutiérrez A, Ullrich R, Hofrichter M, Martínez ÁT (2014). 5-Hydroxymethylfurfural conversion by fungal aryl-alcohol oxidase and unspecific peroxygenase. FEBS J.

[CR7] Carro J, Martínez M, Medina M, Martínez ÁT, Ferreira P (2017). Protein dynamics promote hydride tunnelling in substrate oxidation by aryl-alcohol oxidase. Phys Chem Chem Phys.

[CR8] Chang VT, Crispin M, Aricescu AR, Harvey DJ, Nettleship JE, Fennelly JA, Yu C, Boles KS, Evans EJ, Stuart DI, Dwek RA, Jones EY, Owens RJ, Davis SJ (2007). Glycoprotein structural genomics: solving the glycosylation problem. Structure.

[CR9] Couturier M, Mathieu Y, Li A, Navarro D, Drula E, Haon M, Grisel S, Ludwig R, Berrin JG (2016). Characterization of a new aryl-alcohol oxidase secreted by the phytopathogenic fungus *Ustilago maydis*. Appl Microbiol Biotechnol.

[CR10] de Almeida TP, van Schie MMCH, Ma A, Tieves F, Younes SHH, Fernández-Fueyo E, Arends IWCE, Riul A, Hollmann F (2019). Efficient aerobic oxidation of *trans*-2-hexen-1-ol using the aryl alcohol oxidase from *Pleurotus eryngii*. Adv Synth Catal.

[CR11] Dijkman WP, de Gonzalo G, Mattevi A, Fraaije MW (2013). Flavoprotein oxidases: classification and applications. Appl Microbiol Biotechnol.

[CR12] Fernández IS, Ruíz-Dueñas FJ, Santillana E, Ferreira P, Martínez MJ, Martínez ÁT, Romero A (2009). Novel structural features in the GMC family of oxidoreductases revealed by the crystal structure of fungal aryl-alcohol oxidase. Acta Crystallogr Sect D Biol Crystallogr.

[CR13] Ferreira P, Hernández-Ortega A, Herguedas B, Martínez ÁT, Medina M (2009). Aryl-alcohol oxidase involved in lignin degradation. A mechanistic study based on steady and pre-steady state kinetics and primary and solvent isotope effects with two alcohol substrates. J Biol Chem.

[CR14] Ferreira P, Hernández-Ortega A, Herguedas B, Rencoret J, Gutiérrez A, Martínez MJ, Jiménez-Barbero J, Medina M, Martínez ÁT (2010). Kinetic and chemical characterization of aldehyde oxidation by fungal aryl-alcohol oxidase. Biochem J.

[CR15] Ferreira P, Medina M, Guillén F, Martínez MJ, van Berkel WJH, Martínez AT (2005). Spectral and catalytic properties of aryl-alcohol oxidase, a fungal flavoenzyme acting on polyunsaturated alcohols. Biochem J.

[CR16] Ferreira P, Ruiz-Dueñas FJ, Martínez MJ, Van Berkel WJH, Martínez AT (2006). Site-directed mutagenesis of selected residues at the active site of aryl-alcohol oxidase, an H_2_O_2_-producing ligninolytic enzyme. FEBS J.

[CR17] Forneris F, Orru R, Bonivento D, Chiarelli LR, Mattevi A (2009). ThermoFAD, a Thermofluor®-adapted flavin ad hoc detection system for protein folding and ligand binding. FEBS J.

[CR18] Grote A, Hiller K, Scheer M, Münch R, Nörtemann B, Hempel DC, Jahn D (2005). JCat: A novel tool to adapt codon usage of a target gene to its potential expression host. Nucleic Acids Res.

[CR19] Guillén F, Martínez ÁT, Martínez MJ (1992). Substrate specificity and properties of the aryl-alcohol oxidase from the ligninolytic fungus *Pleurotus eryngii*. Eur J Biochem.

[CR20] Helenius A, Aebi M (2004). Roles of N-linked glycans in the endoplasmic reticulum. Annu Rev Biochem.

[CR21] Hernández-Ortega A, Ferreira P, Merino P, Medina M, Guallar V, Martínez ÁT (2012). Stereoselective hydride transfer by aryl-alcohol oxidase, a member of the GMC superfamily. ChemBioChem.

[CR22] Hernández-Ortega A, Lucas F, Ferreira P, Medina M, Guallar V, Martínez ÁT (2012). Role of active site histidines in the two half-reactions of the aryl-alcohol oxidase catalytic cycle. Biochemistry.

[CR23] Imperiali B, O'Connor SE (1999). Effect of N-linked glycosylatian on glycopeptide and glycoprotein structure. Curr Opin Chem Biol.

[CR24] Jankowski N, Koschorreck K, Urlacher VB (2020). High-level expression of aryl-alcohol oxidase 2 from Pleurotus eryngii in Pichia pastoris for production of fragrances and bioactive precursors. Appl Microbiol Biotechnol.

[CR25] Kadowaki MAS, Higasi PMR, de Godoy MO, de Araujo EA, Godoy AS, Prade RA, Polikarpov I (2020). Enzymatic versatility and thermostability of a new aryl-alcohol oxidase from *Thermothelomyces thermophilus* M77. Biochim Biophys Acta.

[CR26] Kukuruzinska MA, Bergh MLE, Jackson BJ (1987). Protein glycosylation in yeast. Annu Rev Biochem.

[CR27] Kumar S, Nussinov R (2002). Close-range electrostatic interactions in proteins. ChemBioChem.

[CR28] Laemmli UK (1970). Cleavage of structural proteins during assembly of head of bacteriophage-T4. Nature.

[CR29] Li Y, Cirino PC (2014). Recent advances in engineering proteins for biocatalysis. Biotechnol Bioeng.

[CR30] Musafia B, Buchner V, Arad D (1995). Complex salt bridges in proteins: statistical analysis of structure and function. J Mol Biol.

[CR31] Pfaffl MW (2001). A new mathematical model for relative quantification in real-time RT–PCR. Nucleic Acids Res.

[CR32] Ruiz-Dueñas FJ, Ferreira P, Martínez MJ, Martínez AT (2006). In vitro activation, purification, and characterization of *Escherichia coli* expressed aryl-alcohol oxidase, a unique H_2_O_2_-producing enzyme. Protein Expression Purif.

[CR33] Serrano A, Calviño E, Carro J, Sánchez-Ruiz MI, Cañada FJ, Martínez AT (2019). Complete oxidation of hydroxymethylfurfural to furandicarboxylic acid by aryl-alcohol oxidase. Biotechnol Biofuels.

[CR34] Serrano A, Carro J, Martínez ÁT (2020) Reaction mechanisms and applications of aryl-alcohol oxidase. In: Chaiyen P, Tamanoi F (eds) The Enzymes. Flavin-Dependent Enzymes: Mechanisms, Structures and Applications, Vol 47, 2020 edn. Elsevier, pp 167–192 10.1016/bs.enz.2020.05.005 32951823

[CR35] Serrano A, Sancho F, Viña-González J, Carro J, Alcalde M, Guallar V, Martínez ÁT (2019). Switching the substrate preference of fungal aryl-alcohol oxidase: towards stereoselective oxidation of secondary benzyl alcohols. Catal Sci Technol.

[CR36] Tamaru Y, Umezawa K, Yoshida M (2018). Characterization of an aryl-alcohol oxidase from the plant saprophytic basidiomycete *Coprinopsis cinerea* with broad substrate specificity against aromatic alcohols. Biotechnol Lett.

[CR37] Urlacher VB, Koschorreck K (2021). Pecularities and applications of aryl-alcohol oxidases from fungi. Appl Microbiol Biotechnol.

[CR38] van Schie MMCH, de Almeida TP, Laudadio G, Tieves F, Fernández-Fueyo E, Noël T, Arends IWCE, Hollmann F (2018). Biocatalytic synthesis of the green note trans-2-hexenal in a continuous-flow microreactor. Beilstein J Org Chem.

[CR39] Viña-Gonzalez J, Alcalde M (2020). Directed evolution of the aryl-alcohol oxidase: beyond the lab bench. Comput Struct Biotechnol J.

[CR40] Viña-Gonzalez J, Elbl K, Ponte X, Valero F, Alcalde M (2018). Functional expression of aryl-alcohol oxidase in Saccharomyces cerevisiae and Pichia pastoris by directed evolution. Biotechnol Bioeng.

[CR41] Viña-Gonzalez J, Gonzalez-Perez D, Ferreira P, Martínez ÁT, Alcalde M (2015). Focused directed evolution of aryl-alcohol oxidase in Saccharomyces cerevisiae by using chimeric signal peptides. Appl Environ Microbiol.

[CR42] Viña-Gonzalez J, Jimenez-Lalana D, Sancho F, Serrano A, Martínez ÁT, Guallar V, Alcalde M (2019). Structure-guided evolution of aryl alcohol oxidase from *Pleurotus eryngii* for the selective oxidation of secondary benzyl alcohols. Adv Synth Catal.

[CR43] Wang C, Eufemi M, Turano C, Giartosio A (1996). Influence of the carbohydrate moiety on the stability of glycoproteins. Biochemistry.

[CR44] Waterhouse A, Bertoni M, Bienert S, Studer G, Tauriello G, Gumienny R, Heer FT, de Beer TAP, Rempfer C, Bordoli L, Lepore R, Schwede T (2018). SWISS-MODEL: homology modelling of protein structures and complexes. Nucleic Acids Res.

[CR45] Wormald MR, Dwek RA (1999). Glycoproteins: glycan presentation and protein-fold stability. Structure.

[CR46] Wyss DF, Wagner G (1996). The structural role of sugars in glycoproteins. Curr Opin Biotechnol.

