# Supplementary material for: Two adjacent C-terminal mutations enable expression of aryl-alcohol oxidase from Pleurotus eryngii in Pichia pastoris
Source: Appl Microbiol Biotechnol. 2021 Sep 21;105(20):7743–55. doi: 10.1007/s00253-021-11585-4 (PMC8502153; doi:10.1007/s00253-021-11585-4)
Supplement: Supplementary file 1 — Supplementary file1 (PDF 234 KB) [file 253_2021_11585_MOESM1_ESM.pdf]

SUPPLEMENTARY INFORMATION

on

Two adjacent C-terminal mutations enable expression of aryl-alcohol oxidase from *Pleurotus eryngii* in *Pichia pastoris*

Nina Jankowski [a], Vlada B. Urlacher [a], Katja Koschorreck [a]

[a] Institute of Biochemistry, Heinrich-Heine-University Düsseldorf, Universitätsstraße 1, 40225 Düsseldorf, Germany

For correspondence:

Katja Koschorreck

Tel. +49 211 81-10749

Fax +49 211 81-13117

Katja.Koschorreck@hhu.de

ORCID ID 0000-0001-9689-9863

Table S1 Primers used for site-directed mutagenesis of *Pe*AAO1. The triplets introducing the mutations are shown in *italics*

Primer name	Sequence (5' to 3')
***Pe*AAO1_R152G_fw**	CAACAATTCGTT*GGT*AAGAACGAAATGG
***Pe*AAO1_R152G_rev**	CCATTTCGTTCTT*ACC*AACGAATTGTTG
***Pe*AAO1_T265I_fw**	GGTTAACTCTGGT*ATC*ACTAACGGTTTGC
***Pe*AAO1_T265I_rev**	GCAAACCGTTAGT*GAT*ACCAGAGTTAACC
***Pe*AAO1_D361N_fw**	GACAACATCTTCAGA*AAC*TCTTCTGAATTTAACG
***Pe*AAO1_D361N_rev**	CGTTAAATTCAGAAGA*GTT*TCTGAAGATGTTGTCG
***Pe*AAO1_V367A_fw**	GACTCTTCTGAATTTAAC*GCT*GACTTGGACCAATGG
***Pe*AAO1_V367A_rev**	CCATTGGTCCAAGTC*AGC*GTTAAATTCAGAAGAGTC
***Pe*AAO1_D512N_fw**	GAGACCCAACT*AAC*GACGCTGCTATCGAATC
***Pe*AAO1_D512N_rev**	GATTCGATAGCAGCGTC*GTT*AGTTGGGTCTC
***Pe*AAO1_K583E_fw**	CCAATCTACTTGGTTGGT*GAA*CAAGGTGCTGACTTG
***Pe*AAO1_K583E_rev**	CAAGTCAGCACCTTG*TTC*ACCAACCAAGTAGATTGG
***Pe*AAO1_Q584R_fw**	CTACTTGGTTGGTAAG*AGA*GGTGCTGACTTGATC
***Pe*AAO1_Q584R_rev**	GATCAAGTCAGCACC*TCT*CTTACCAACCAAGTAG
***Pe*AAO1_K583E/Q584R_fw**	TTGGT*GAA*AGAGGTGCTGACTTGATCAAGGCTG
***Pe*AAO1_K583E/Q584R_rev**	CAGCACCTCT*TTC*ACCAACCAAGTAGATTGG

```
PeAAO1 WT      MSFGALRQLLLIACLALPSLAATNLPTADFDYVVVGAGNAGNVVAARLTEDPDVSVLVLE    60
PeAAO1 ER      MSFGALRQLLLIACLALPSLAATNLPTADFDYVVVGAGNAGNVVAARLTEDPDVSVLVLE    60
PeAAO1 NER     MSFGALRQLLLIACLALPSLAATNLPTADFDYVVVGAGNAGNVVAARLTEDPDVSVLVLE    60
PeAAO1 AER     MSFGALRQLLLIACLALPSLAATNLPTADFDYVVVGAGNAGNVVAARLTEDPDVSVLVLE    60
PeAAO2 WT      MSFGALRQLLLIACLALPSLAATNLPTADFDYVVVGAGNAGNVVAARLTEDPDVSVLVLE    60
               ************************************************************

PeAAO1 WT      AGVSDENVLGAEAPLLAPGLVPNSIFDWNYTTTAQAGYNGRSIAYPRGRMLGGSSSVHYM    120
PeAAO1 ER      AGVSDENVLGAEAPLLAPGLVPNSIFDWNYTTTAQAGYNGRSIAYPRGRMLGGSSSVHYM    120
PeAAO1 NER     AGVSDENVLGAEAPLLAPGLVPNSIFDWNYTTTAQAGYNGRSIAYPRGRMLGGSSSVHYM    120
PeAAO1 AER     AGVSDENVLGAEAPLLAPGLVPNSIFDWNYTTTAQAGYNGRSIAYPRGRMLGGSSSVHYM    120
PeAAO2 WT      AGVSDENVLGAEAPLLAPGLVPNSIFDWNYTTTAQAGYNGRSIAYPRGRMLGGSSSVHYM    120
               ************************************************************
                                              R152
PeAAO1 WT      VMMRGSTEDFDRYAAVTGDEGWNWDNIQQFVRKNEMVVPPADNHNTSGEFIPAVHGTNGS    180
PeAAO1 ER      VMMRGSTEDFDRYAAVTGDEGWNWDNIQQFVRKNEMVVPPADNHNTSGEFIPAVHGTNGS    180
PeAAO1 NER     VMMRGSTEDFDRYAAVTGDEGWNWDNIQQFVRKNEMVVPPADNHNTSGEFIPAVHGTNGS    180
PeAAO1 AER     VMMRGSTEDFDRYAAVTGDEGWNWDNIQQFVRKNEMVVPPADNHNTSGEFIPAVHGTNGS    180
PeAAO2 WT      VMMRGSTEDFDRYAAVTGDEGWNWDNIQQFVGKNEMVVPPADNHNTSGEFIPAVHGTNGS    180
               ******************************* ****************************

PeAAO1 WT      VSISLPGFPTPLDDRVLATTQEQSEEFFFNPDMGTGHPLGISWSIASVGNGQRSSSSTAY    240
PeAAO1 ER      VSISLPGFPTPLDDRVLATTQEQSEEFFFNPDMGTGHPLGISWSIASVGNGQRSSSSTAY    240
PeAAO1 NER     VSISLPGFPTPLDDRVLATTQEQSEEFFFNPDMGTGHPLGISWSIASVGNGQRSSSSTAY    240
PeAAO1 AER     VSISLPGFPTPLDDRVLATTQEQSEEFFFNPDMGTGHPLGISWSIASVGNGQRSSSSTAY    240
PeAAO2 WT      VSISLPGFPTPLDDRVLATTQEQSEEFFFNPDMGTGHPLGISWSIASVGNGQRSSSSTAY    240
               ************************************************************
                                       T265
PeAAO1 WT      LRPAQSRPNLSVLINAQVTKLVNSGTTNGLPAFRCVEYAEQEGAPTTTVCAKKEVVLSAG    300
PeAAO1 ER      LRPAQSRPNLSVLINAQVTKLVNSGTTNGLPAFRCVEYAEQEGAPTTTVCAKKEVVLSAG    300
PeAAO1 NER     LRPAQSRPNLSVLINAQVTKLVNSGTTNGLPAFRCVEYAEQEGAPTTTVCAKKEVVLSAG    300
PeAAO1 AER     LRPAQSRPNLSVLINAQVTKLVNSGTTNGLPAFRCVEYAEQEGAPTTTVCAKKEVVLSAG    300
PeAAO2 WT      LRPAQSRPNLSVLINAQVTKLVNSGITNGLPAFRCVEYAEQEGAPTTTVCAKKEVVLSAG    300
               ************************ ***********************************

PeAAO1 WT      SVGTPILLQLSGIGDENDLSSVGIDTIVNNPSVGRNLSDHLLLPAAFFVNSNQTFDNIFR    360
PeAAO1 ER      SVGTPILLQLSGIGDENDLSSVGIDTIVNNPSVGRNLSDHLLLPAAFFVNSNQTFDNIFR    360
PeAAO1 NER     SVGTPILLQLSGIGDENDLSSVGIDTIVNNPSVGRNLSDHLLLPAAFFVNSNQTFDNIFR    360
PeAAO1 AER     SVGTPILLQLSGIGDENDLSSVGIDTIVNNPSVGRNLSDHLLLPAAFFVNSNQTFDNIFR    360
PeAAO2 WT      SVGTPILLQLSGIGDENDLSSVGIDTIVNNPSVGRNLSDHLLLPAAFFVNSNQTFDNIFR    360
               ************************************************************
               D361  V367
PeAAO1 WT      DSSEFNVDLDQWTNTRTGPLTALIANHLAWLRLPSNSSIFQTFPDPAAGPNSAHWETIFS    420
PeAAO1 ER      DSSEFNVDLDQWTNTRTGPLTALIANHLAWLRLPSNSSIFQTFPDPAAGPNSAHWETIFS    420
PeAAO1 NER     NSSEFNVDLDQWTNTRTGPLTALIANHLAWLRLPSNSSIFQTFPDPAAGPNSAHWETIFS    420
PeAAO1 AER     DSSEFNADLDQWTNTRTGPLTALIANHLAWLRLPSNSSIFQTFPDPAAGPNSAHWETIFS    420
PeAAO2 WT      NSSEFNADLDQWTNTRTGPLTALIANHLAWLRLPSNSSIFQTFPDPAAGPNSAHWETIFS    420
               :*****.*****************************************************

PeAAO1 WT      NQWFHPAIPRPDTGSFMSVTNALISPVARGDIKLATSNPFDKPLINPQYLSTEFDIFTMI    480
PeAAO1 ER      NQWFHPAIPRPDTGSFMSVTNALISPVARGDIKLATSNPFDKPLINPQYLSTEFDIFTMI    480
PeAAO1 NER     NQWFHPAIPRPDTGSFMSVTNALISPVARGDIKLATSNPFDKPLINPQYLSTEFDIFTMI    480
PeAAO1 AER     NQWFHPAIPRPDTGSFMSVTNALISPVARGDIKLATSNPFDKPLINPQYLSTEFDIFTMI    480
PeAAO2 WT      NQWFHPAIPRPDTGSFMSVTNALISPVARGDIKLATSNPFDKPLINPQYLSTEFDIFTMI    480
               ************************************************************
                                              D512
PeAAO1 WT      QAVKSNLRFLSGQAWADFVIRPFDPRLRDPTDDAAIESYIRDNANTIFHPVGTASMSPRG    540
PeAAO1 ER      QAVKSNLRFLSGQAWADFVIRPFDPRLRDPTDDAAIESYIRDNANTIFHPVGTASMSPRG    540
PeAAO1 NER     QAVKSNLRFLSGQAWADFVIRPFDPRLRDPTDDAAIESYIRDNANTIFHPVGTASMSPRG    540
PeAAO1 AER     QAVKSNLRFLSGQAWADFVIRPFDPRLRDPTDDAAIESYIRDNANTIFHPVGTASMSPRG    540
PeAAO2 WT      QAVKSNLRFLSGQAWADFVIRPFDPRLRDPTNDAAIESYIRDNANTIFHPVGTASMSPRG    540
               *******************************:****************************
                                                         K583/Q584
PeAAO1 WT      ASWGVVDPDLKVKGVDGLRIVDGSILPFAPNAHTQGPIYLVGKQGADLIKADQ    593
PeAAO1 ER      ASWGVVDPDLKVKGVDGLRIVDGSILPFAPNAHTQGPIYLVGERGADLIKADQ    593
PeAAO1 NER     ASWGVVDPDLKVKGVDGLRIVDGSILPFAPNAHTQGPIYLVGERGADLIKADQ    593
PeAAO1 AER     ASWGVVDPDLKVKGVDGLRIVDGSILPFAPNAHTQGPIYLVGERGADLIKADQ    593
PeAAO2 WT      ASWGVVDPDLKVKGVDGLRIVDGSILPFAPNAHTQGPIYLVGERGADLIKADQ    593
               ******************************************::*********
```

Fig. S1 Protein sequence alignment of *Pe*AAO1 and *Pe*AAO2 wild-type (Accession numbers AAC72747 and ADD14021) and of *Pe*AAO1 variants ER, NER and AER. Orange boxes with red labeling refer to the seven differing amino acid positions in *Pe*AAO1 and *Pe*AAO2 wild-type. Amino acids present in *Pe*AAO2 are shown in bold black. Mutated amino acids in *Pe*AAO1 variants ER, NER and AER are shown in bold blue

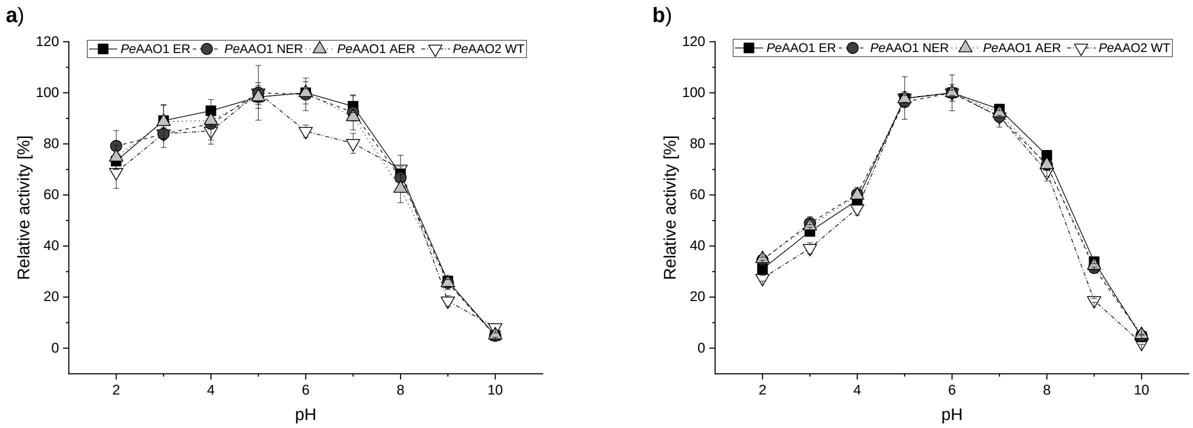

Fig. S2 pH activity profile of *Pe*AAO1 variants and *Pe*AAO2 wild-type towards **a)** *p*-anisyl alcohol and **b)** veratryl alcohol. 5 mM substrate and 100 mM Britton-Robinson buffer were used. *Pe*AAO1 ER: black squares, solid line; *Pe*AAO1 NER: dark grey circles, dashed line; *Pe*AAO1 AER: light grey triangles, dotted line; *Pe*AAO2 wild-type: white triangles, dashed-dotted line. Highest activity was set to 100 %. Data for *Pe*AAO2 wild-type taken from Jankowski et al. 2020

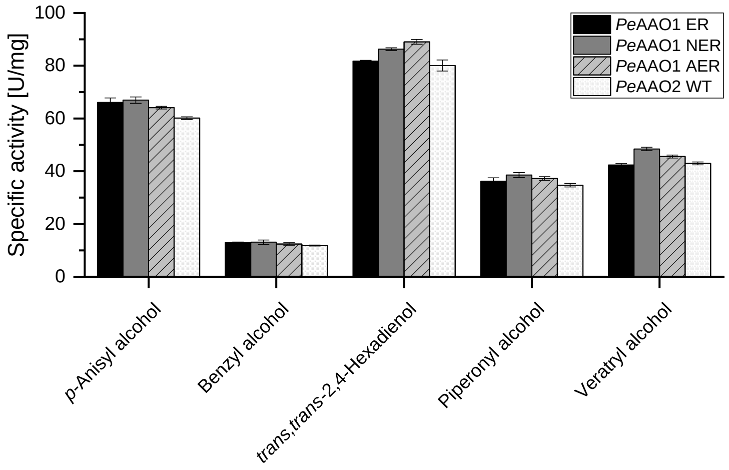

Fig. S3 Specific activities [U/mg] of *Pe*AAO1 variants and *Pe*AAO2 wild-type towards several substrates. 5 mM of each substrate was used in 100 mM sodium phosphate buffer pH 6. *Pe*AAO1 ER: black, filled; *Pe*AAO1 NER: grey, filled; *Pe*AAO1 AER: grey, striped; *Pe*AAO2 wild-type: white, dotted

References

Jankowski N, Koschorreck K, Urlacher VB (2020) High-level expression of aryl-alcohol oxidase 2 from *Pleurotus eryngii* in *Pichia pastoris* for production of fragrances and bioactive precursors. Appl Microbiol Biotechnol 104(21):9205-9218. doi:10.1007/s00253-020-10878-4
